# Decomposing functional trait associations in a Chinese subtropical forest

**DOI:** 10.1371/journal.pone.0175727

**Published:** 2017-04-18

**Authors:** Xuefei Li, Kequan Pei, Marc Kéry, Pascal A. Niklaus, Bernhard Schmid

**Affiliations:** 1 Department of Evolutionary Biology and Environmental Studies, University of Zurich, Zurich, Switzerland; 2 Laboratory of Quantitative Vegetation Ecology, Institute of Botany, Chinese Academy of Sciences, Xiangshan, Haidian District, Beijing, P.R. China; 3 Swiss Ornithological Institute, Sempach, Switzerland; Chinese Academy of Forestry, CHINA

## Abstract

Functional traits, properties of organisms correlated with ecological performance, play a central role in plant community assembly and functioning. To some extents, functional traits vary in concert, reflecting fundamental ecological strategies. While “trait syndromes” characteristic of e.g. fast-growing, early-successional vs. competitive, late-successional species are recognized in principle, less is known about the environmental and genetic factors at the source of trait variation and covariation within plant communities. We studied the three leaf traits leaf half-life (LHL), leaf mass per area (LMA) and nitrogen concentration in green leaves (N_green_) and the wood trait wood density (WD) in 294 individuals belonging to 45 tree or shrub species in a Chinese subtropical forest from September 2006 to January 2009. Using multilevel ANOVA and decomposition of sums of products, we estimated the amount of trait variation and covariation among species (mainly genetic causes), i.e. plant functional type (deciduous vs. evergreen species), growth form (tree vs. shrub species), family/genus/species differences, and within species (mainly environmental causes), i.e. individual and season. For single traits, the variation between functional types and among species within functional types was large, but only LMA and N_green_ varied significantly among families and thus showed phylogenetic signal. Trait variation among individuals within species was small, but large temporal variation due to seasonal effects was found within individuals. We did not find any trait variation related to soil conditions underneath the measured individuals. For pairs of traits, variation between functional types and among species within functional types was large, reflecting a strong evolutionary coordination of the traits, with LMA, LHL and WD being positively correlated among each other and negatively with N_green_. This integration of traits was consistent with a putative stem-leaf economics spectrum ranging from deciduous species with thin, high-nitrogen leaves and low-density wood to evergreen species with thick, low-nitrogen leaves and dense wood and was not influenced by phylogenetic history. Trait coordination within species was weak, allowing individual trees to deviate from the interspecific trait coordination and thus respond flexibly to environmental heterogeneity. Our findings suggest that within a single woody plant community variation and covariation in functional traits allows a large number of species to co-exist and cover a broad spectrum of multivariate niche space, which in turn may increase total resource extraction by the community and community functioning.

## Introduction

The amount of variation in functional traits found among higher plants is enormous. For example, leaf nitrogen concentrations, leaf mass per area and leaf life-span vary by up to two orders of magnitude across communities [1, 2]. Such a wide trait range may appear surprising in the light of plants requiring essentially the same set of basic resources. However, species acquire these resources in different ways and require them in different quantities [3].

Across communities, trait variation is related to the functional strategies of species that allow them to perform under the environmental conditions prevailing in their habitat. This “environmental filtering” [4, 5] limits the range of traits with which a species can successfully perform under given environmental condition. Within communities, however, competitive interactions prevent coexisting species from being too similar (“limiting similarity” [2, 4, 6–8]) unless their competitive strengths are so similar that they may coexist for a long time (“neutral theory of biodiversity” [9]).

In general, multiple traits do not vary independently in integrated phenotypes but form patterns of covariance. Experimental and theoretical studies have suggested that this coordination of traits can emerge from many sources, including developmental (e.g. allometric) constraints, genetic constraints, and resource-investment tradeoffs. As a consequence, typical sets of correlated traits manifest as "trait syndromes" that define the ecological strategies of species and thus their coexistence within a community and the sorting of species along broad environmental gradients.

Analyses of leaf trait coordination indicate that many leaf traits vary in concert along a multidimensional optimum [2]. This so called leaf economics spectrum is defined by a continuum between fast-growing species with inexpensive, thin, short-lived leaves providing a rapid return of investments in terms of leaf carbon and slow-growing species with thick, long-lived leaves with comparably low photosynthetic capacity that pay back their structural costs over longer time scales [2, 10]. The leaf economics spectrum may be extended to include wood traits because woody tissues are likely to face similar physiological, structural and defensive trade-offs as leaves do [11]. The resulting stem-leaf economics spectrum reflects a coordination of investment in leaf and wood tissue as a plant strategy and could explain why fast-growing species tend to have thin leaves and low wood density, and vice versa, but evidence for this currently is inconsistent [11, 12]. While trait correlations such as the ones found in the leaf economics spectrum follow a broad general pattern, this relation varies to some extent across species and habitats. In fact, such correlations can be considered as (higher order) traits subject to similar ecological constraints and selective forces as individual traits.

Variation in single traits and correlations of multiple traits can typically be attributed to genetic and environmental causes [13]. Within communities, interspecific variation in functional traits will have a larger contribution of genetic causes than between communities where plastic responses to environmental variation are more important. However, environmental variation also occurs within a single community due to smaller-scale spatial heterogeneity in environmental conditions but also due to variation in time, in particular between seasons.

Apart from promoting compositional stability, variation in functional traits within a community is important because it might contribute to ecosystem functioning. An ever increasing number of biodiversity–ecosystem functioning studies indicate an improved and more stable functioning of more diverse ecosystems [14–16]. These effects are ultimately related to functionally more diverse sets of species using a larger total amount of resources [17] or being less susceptible to detrimental effects of pathogens or herbivores [18]. Understanding trait variation and covariation and their origins is thus not only relevant from a community assembly perspective but also for the development of our understanding of biodiversity–ecosystem functioning relationships.

Here, we recorded traits on 294 individuals of 45 woody species in a species-rich subtropical forest in southwestern China. As traits we chose leaf half-life (LHL), leaf mass per area (LMA), nitrogen concentration of green leaves (N_green_) and wood density (WD). The leaf traits chosen integrate physiological constraints and trade-offs with respect to light interception, photosynthetic capacity and carbon return on investment (and thus relative growth rates; [1, 2, 10]. WD is of central importance because it is associated with hydraulic properties and rates of plant growth and mortality [11, 19]. We analyzed both patterns of variation and covariation among the measured functional traits. We decomposed trait variances and covariances into between-species and within-species components. We considered the between-species components as largely of genetic origin and were interested in the fraction of trait variation and covariation that could be attributed to plant functional type (deciduous vs. evergreen species), growth form (tree vs. shrub species) and taxonomy (family, genus, species). The within-species variation and covariation was considered as largely environment-driven and was analyzed in dependence of individual and season.

## Materials and methods

### Study site and design

The present study was conducted in a secondary broad-leaved and coniferous mixed subtropical forest in Dujiangyan, Sichuan province, SW China. No specific permissions were required for the field work as the forest is for the public. No endangered or protected species were included in this study.

With the rising slopes of the Qinghai-Tibet plateau to the west and the fertile agricultural plains of the Sichuan basin to the east, the Dujiangyan area is one of eleven biodiversity hotspots in China [20]. The climatic conditions are typically subtropical with a dry winter (November–April) and a warm and rainy summer (May–October). The annual average temperature is 15°C, with an average July temperature of 25°C. The mean annual precipitation is ~1300 mm, and the annual average relative air humidity exceeds 80% [20].

The study site covers approximately 22 ha (31°03’43”–31°04’03” N, 103°42’55”–103°43’52” E), and elevation ranges from 693 to 830 m above sea level. Previous vegetation surveys indicated 158 species of broad-leaved evergreen, deciduous and coniferous trees and shrubs in the forest (Du, unpublished data). The forest canopy is dominated by *Castanopsis fargesii*, *Betula luminifera*, *Quercus serrata* and *Quercus variabilis* (all nomenclature in this paper follows “Flora of China”, [21]. *Camellia oleifera* and *Eurya alata* are the most abundant shrub species. The dominant soil types are Leptosols, Regosols and Cambisols from loess-like material, with an average nitrogen concentration of 0.3% and organic matter concentration of ca. 9% [20].

We selected 45 woody angiosperm species representing 20 families (Table 1). Species were classified according to functional type (20 deciduous and 25 evergreen species) and growth form (34 tree and 11 shrub species). We assessed leaf mass per area, leaf nitrogen content and wood density of all individuals a first time in April (spring) and again in September (fall) 2007.

**Table 1:** List of the 45 species used in this study with functional type, growth form and number of individuals measured (n). Species names follow the”Dujiangyan higher plant name list”provided by the local herbarium of West China Subalpine Botanical Garden, Institute of Botany, Chinese Academy of Sciences. Note that *Kalopanax pictus* is synonymous to *Kalopanax septemlobus*. For author names of species see Flora of China [21].

Family	Genus	Species	Functional type	Growth form	n
Anacardiaceae	*Choerospondias*	*axillaris*	deciduous	tree	5
	*Pistacia*	*chinensis*	deciduous	tree	16
	*Rhus*	*chinensis*	deciduous	tree	9
		*punjabensis*	deciduous	tree	1
	*Toxicodendron*	*succedaneum*	deciduous	tree	9
Aquifoliaceae	*Ilex*	*chinensis*	evergreen	tree	6
		*szechwanensis*	evergreen	shrub	10
Araliaceae	*Aralia*	*chinensis*	deciduous	shrub	5
	*Kalopanax*	*pictus*	deciduous	tree	6
Betulaceae	*Betula*	*luminifera*	deciduous	tree	8
Cornaceae	*Cornus*	*controversa*	deciduous	tree	5
Ebenaceae	*Diospyros*	*kaki*	deciduous	tree	1
Elaeocarpaceae	*Elaeocarpus*	*japonicus*	evergreen	tree	8
Euphorbiaceae	*Mallotus*	*philippensis*	deciduous	tree	5
		*tenuifolius*	evergreen	tree	5
Fagaceae	*Castanopsis*	*carlesii*	evergreen	tree	4
		*fargesii*	evergreen	tree	6
		sp.	evergreen	tree	4
	*Cyclobalanopsis*	*glauca*	evergreen	tree	14
	*Lithocarpus*	*hancei*	evergreen	tree	10
	*Quercus*	*serrata*	deciduous	tree	12
		*variabilis*	deciduous	tree	11
Juglandaceae	*Platycarya*	*strobilacea*	deciduous	tree	6
	*Pterocarya*	*stenoptera*	deciduous	tree	4
Lauraceae	*Cinnamomm*	*bodinieri*	evergreen	tree	8
	*Lindera*	*communis*	evergreen	shrub	5
	*Machilus*	*pingii*	evergreen	tree	5
Moraceae	*Ficus*	*henryi*	evergreen	shrub	5
		*heterophylla*	deciduous	tree	1
Myrsinaceae	*Myrsine*	*africana*	evergreen	shrub	8
Olacaceae	*Schoepfia*	*jasminodora*	deciduous	tree	2
Pittosporaceae	*Pittosporum*	*podocarpum*	evergreen	shrub	7
		sp.	evergreen	shrub	3
Rosaceae	*Photinia*	*davidsoniae*	evergreen	tree	5
	*Pyracantha*	*fortuneana*	evergreen	shrub	5
Rutaceae	*Zanthoxylum*	*ovalifolium*	deciduous	tree	6
Symplocaceae	*Symplocos*	*anomala*	deciduous	tree	5
		*cochinchinensis* var. *laurina*	evergreen	tree	10
		*paniculata*	evergreen	tree	4
		*stellaris*	evergreen	tree	8
		*sumuntia*	evergreen	tree	10
Theaceae	*Camellia*	*oleifera*	evergreen	shrub	13
	*Eurya*	*alata*	evergreen	shrub	6
		*nitida*	evergreen	shrub	7
Ulmaceae	*Celtis*	*vardervoetiana*	deciduous	tree	1

### Leaf mass per area and leaf nitrogen content

We sampled 5–8 fully expanded current season leaves from the edge of the leaf crown of each individual. Leaves were collected at middle crown height except for trees taller than 15 m in which we sampled closer to the ground for logistic reasons. The cumulative area of the leaves excluding petioles was determined (LI-3100C Area Meter, LI-COR, Lincoln, NE) and the samples weighed after drying (60°C, 72 h). Leaf nitrogen concentration (N_green,_ % of dry mass) was determined by dry combustion (CHNS–932 elemental analyzer, LECO, St. Joseph, MI).

### Leaf demography and leaf half-life

Leaf half life (LHL) was determined by leaf demographic analysis in 8 deciduous and 24 evergreen species (90 tree individuals in total for which we could reach at least one healthy sunlit branch). In April 2007, we marked a cohort of 5–20 young leaves on the terminal section of one or (if possible) two healthy sunlit branches per tree. These branches were revisited in August 2007, March 2008, August 2008 and January 2009 and a new mark placed at the tip of each branch. At each census, the number of live leaves between adjacent marks was determined. Leaves on side branches that developed during the census were not taken into account.

We then determined LHL as the time required for half of the leaves to abscise. Given leaf numbers N_1_ and N_2_ at the start and end of each interval, and the length of the censored interval ΔT = T_2_–T_1_, leaf half life equals LHL = (T_2_–T_1_) · ln(1/2) / ln(N_2_/N_1_). These calculations rest on the assumption of an exponential decrease of the number of leaves during the censored interval, i.e. a constant abscission rate [22]. LHL estimates were obtained for each leaf cohort and census interval, resulting in a maximum of 10 estimates per tree (2 separate branches, with 5 leaf cohorts corresponding to 5 intervals). Strictly, the age of each leaf is not known when the cohort is marked the first time. However, we did not find significant differences between LHL estimates from the first census (assuming the leaves had age zero when the cohort was established) and the following censuses. Furthermore, the first cohort produced the most stable LHL estimates due to the large number of leaves, and we therefore only included these LHL estimates in the final data analysis.

### Wood density

We collected 5 cm long cores (excluding bark) with an increment borer (diameter 5.15 mm, Haglöf, Sweden). The fresh volume of the cores was determined immediately after sampling by water displacement. The cores were then dried (60°C, 72 h) and weighed and wood density calculated as mass per fresh volume.

### Soil organic carbon and nitrogen

In August 2009, a 3 cm diameter × 10 cm depth soil core was collected underneath the leaf crown of a subset of 132 tree individuals (representing 12 deciduous and 24 evergreen species). The soil samples were dried, ground and analyzed for organic C and N contents by dry combustion (FOSS 2200 elemental analyser, Foss Tecator AB, Sweden).

## Data analysis

We first analyzed trait variances and covariances using linear models, quantifying the relative importance of functional type, growth form, family, species, individual and season. We then tested for evidence of phylogenetic conservatism of traits or trait covariances.

### Analysis of trait variances

Trait variances were analyzed by fitting hierarchical linear models with terms for the sources of variance in which we were interested. These models were fitted in a Bayesian framework, using Markov chain Monte Carlo (MCMC) simulation methods in WinBUGS [23, 24], run from R 2.12.2 (http://www.r-project.org) via the R2WinBUGS interface [25]. Trait y of individual *i* = 1…*n* was modelled as y_i_ ~ N(μi,σ_i_^2^), with μ_i_ = α_0_+α_FT(i)_+α_GF(i)_+α_season(i)_+β_fam(i)_+β_spec(i)_+β_ind(i)_. α_0_ is the grand mean, α_FT(i)_, α_GF(i)_ and α_season(i)_ are the fixed effects of functional type, growth form and season (except for LHL), and β_fam(i)_, β_spec(i)_ and β_ind(i)_ are the random effects of family, species and individual. These random effects defined the different error levels and were all assumed to be normally distributed as β_fam_ = N(0, σ^2^_fam_), β_spec_ = N(0, σ^2^_spec_) and β_ind_ = N(0, σ^2^_ind_). The residual term corresponded to the variation between seasons nested within individuals (except for LHL where it corresponded to the variation among individuals).

We used standard deviations of each fixed and random effect as a measure for the importance of each source of trait variation [26, 27]. For fixed-effects (α_FT_, α_GF_ and α_season_) we computed the finite-population standard deviation as the standard deviation of the coefficients in the population of predicted values [28]. Sample size was 397 for LMA, 90 for LHL, 333 for N_green_ and 241 for WD.

We used conventional vague priors for all parameters, i.e. *N*(0, 1000), for the grand mean α_0_, U(–3,3) for the regression parameters underlying the fixed effects α_FT_, α_GF_ and α_season_, and U(0,10) for the square root of the variance components σ^2^_fam_, σ^2^_spec_, σ^2^_ind_ and σ^2^_y_. Bayesian posterior estimates using such vague priors resemble estimates from maximum likelihood methods [29, 30] but are exact rather than approximate because they account for the full uncertainty in the modeled system [28]. For each response, we ran three Markov chains until convergence was achieved according to the “Rhat” test statistic (Rhat < 1.2). Posterior means and standard deviations are summarized in S1 Table.

It should be noted that due to the low replication within species, we only calculated a pooled within-species variance component (*β*_*ind*_) and that for this pooled component the replication was quite high because the number of species in our analysis was high. We aimed at analyzing as many species as possible and thus spread our sampling effort across a large number of species at the expense of a low replication within species.

### Analyses of trait covariances

The contributions of functional type, growth form, family, species, individual and season to trait covariances was analyzed in an analogues way to the analysis of single traits described above. In brief, the sum of products of each trait pair was decomposed into contributions of the respective terms, analogues to the decomposition of sum of squares in analysis of variance. The percentage of sum of products explained by each term indicates the percentage of total covariance explained by the corresponding term [11, 31]. This analysis was implemented in GenStat (11th edition, VSN International Ltd, UK). All of the traits were log-transformed as their distributions were right-skewed. Log transformation of traits also linearizes the power relationships between traits that can be expected for allometric reasons.

### Phylogenetic signals in patterns of variance and covariance

Phylogenetic signals in single traits were tested using Blomberg’s K [32] which is the ratio of the mean squared error of the tip data divided by the mean squared error of the data calculated using the variance-covariance matrix derived from the phylogenic tree; this observed ratio is then standardized by the ratio expected under Brownian-motion evolution [32]. A value around 1 typifies Brownian evolution characteristics while a value close to 0 indicates the absence of a phylogenetic signal. The significance of K-values was tested by repeated (n = 1000) randomization of traits among species and calculating 95% confidence intervals. Our analyses are based on the “supertree” from Phylomatic [33] which compiles published angiosperm phylogenies.

Evolutionary divergence patterns in trait-pairs was tested with phylogenetically independent contrasts (PICs) [34]. PICs for each trait pair were calculated using package “ape” in R [35].

## Results

### Individual traits

**Fig 1:**
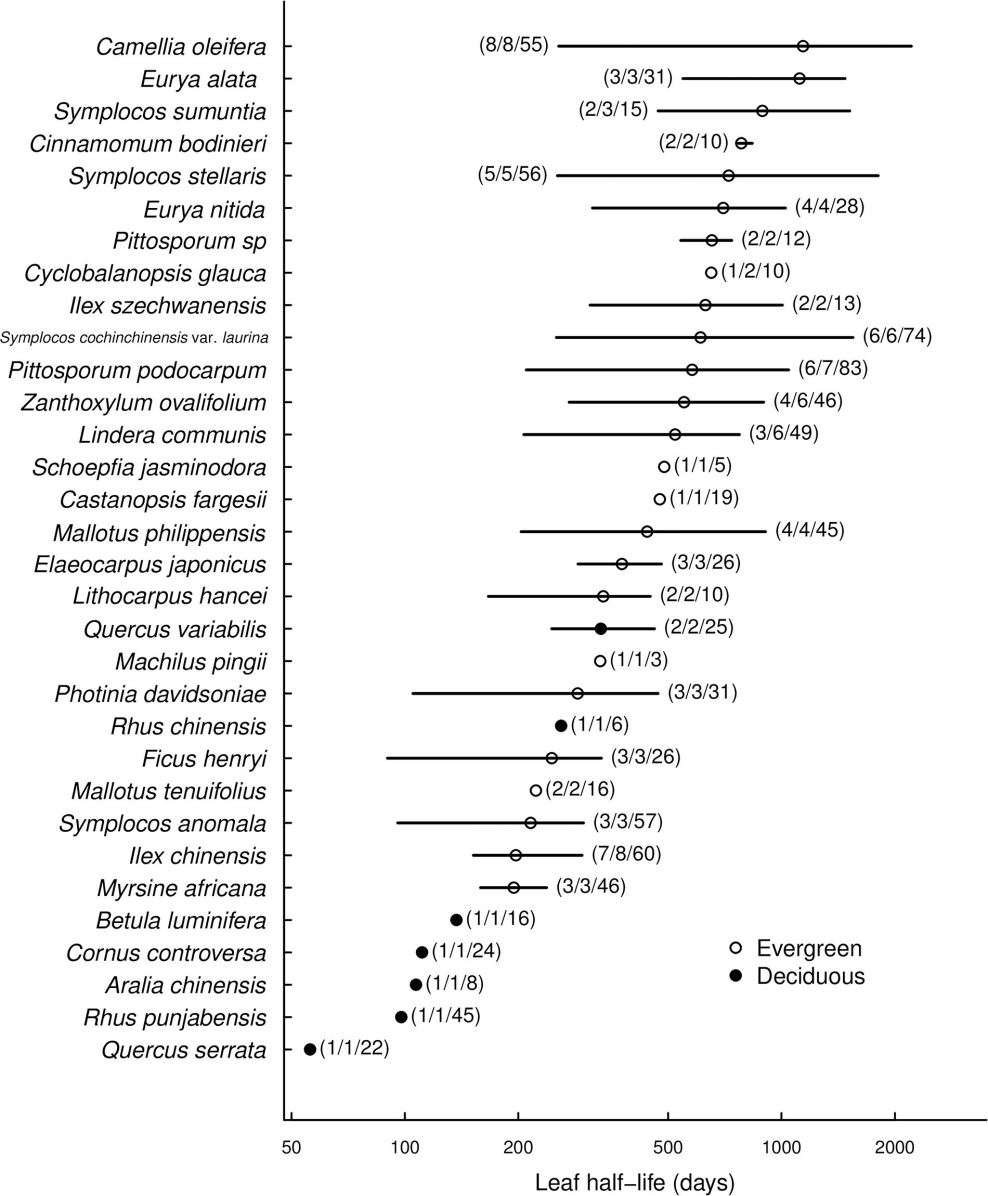
Leaf half-life (LHL) for a subset of 32 species. Points indicate species means and lines indicate the range of data. The numbers in brackets indicate numbers of individuals, branches and leaves used in estimating LHL. Circles represent evergreen species and solid points represent deciduous species. For species taxonomy see Table 1.

**Fig 2:**
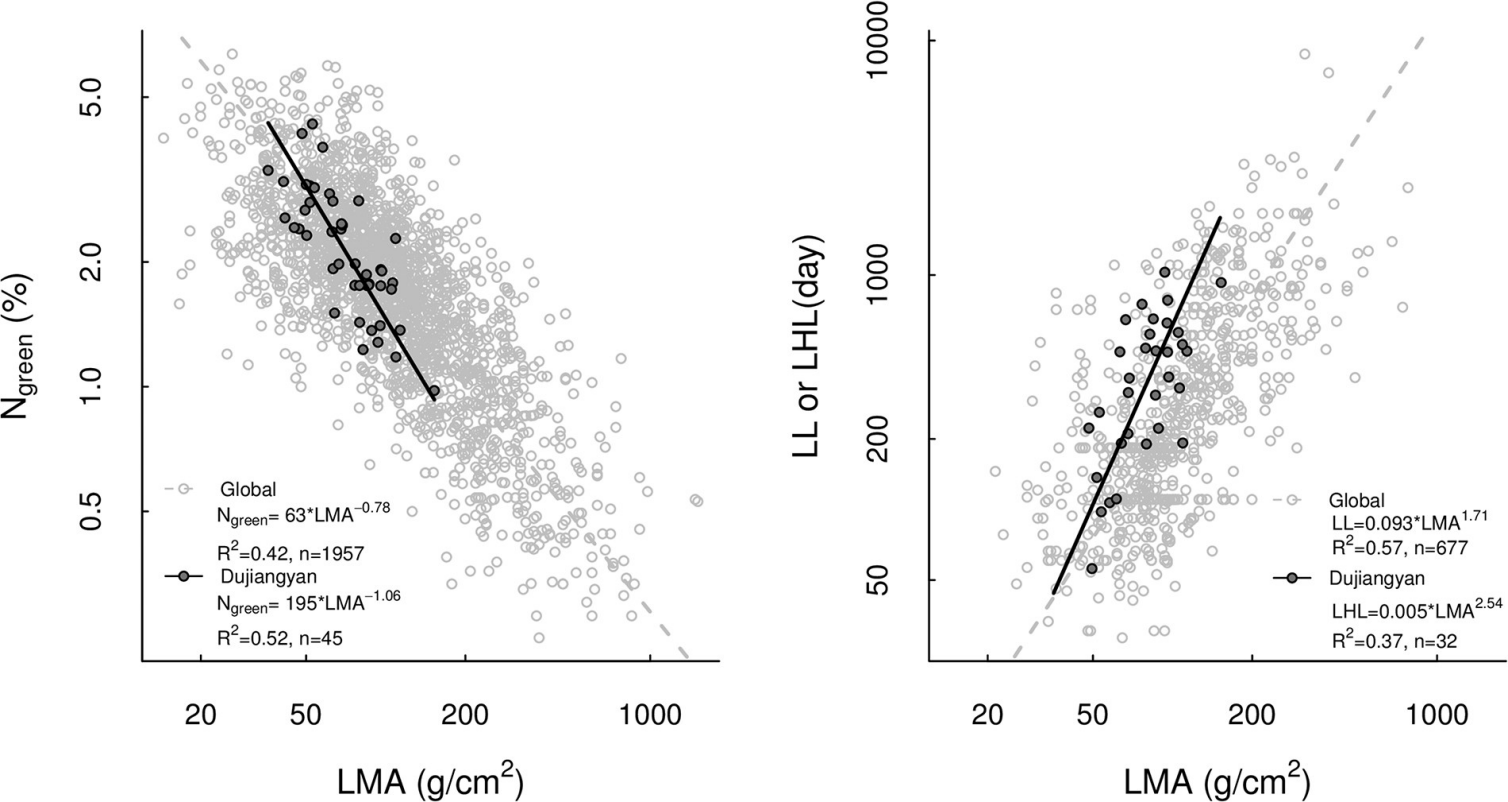
Relationships between leaf mass per area (LMA), nitrogen concentration in green leaves (N_green_) and leaf half-life (LHL) for the forest community at Dujiangyan and a global dataset [2]. Light grey circles represent species means in the global dataset, black points represent species means from the present study.

Overall, LMA varied 5-fold (38–156 g/cm^2^), N_green_ varied 4-fold (1.0–4.3%), LHL varied 20-fold (56–1140 days; Fig 1) and WD varied 2-fold (0.36–0.76 g/cm^3^) among species. In comparison, LMA varied 7-fold (27–198 g/cm^2^), N_green_ varied 6-fold (0.8–5.0%), LHL varied 40-fold (56–2211 days) and WD varied 3-fold (0.25–1.2 g/cm^3^) among individuals (S2 Table). The range of leaf trait values covered in our study was remarkable compared to the global dataset [2], with traits covering large percentile ranges of the global database (Fig 2; LMA: 7–75%, N_green_: 16%−97.5%, LHL: 2.5%−90%).

**Fig 3:**
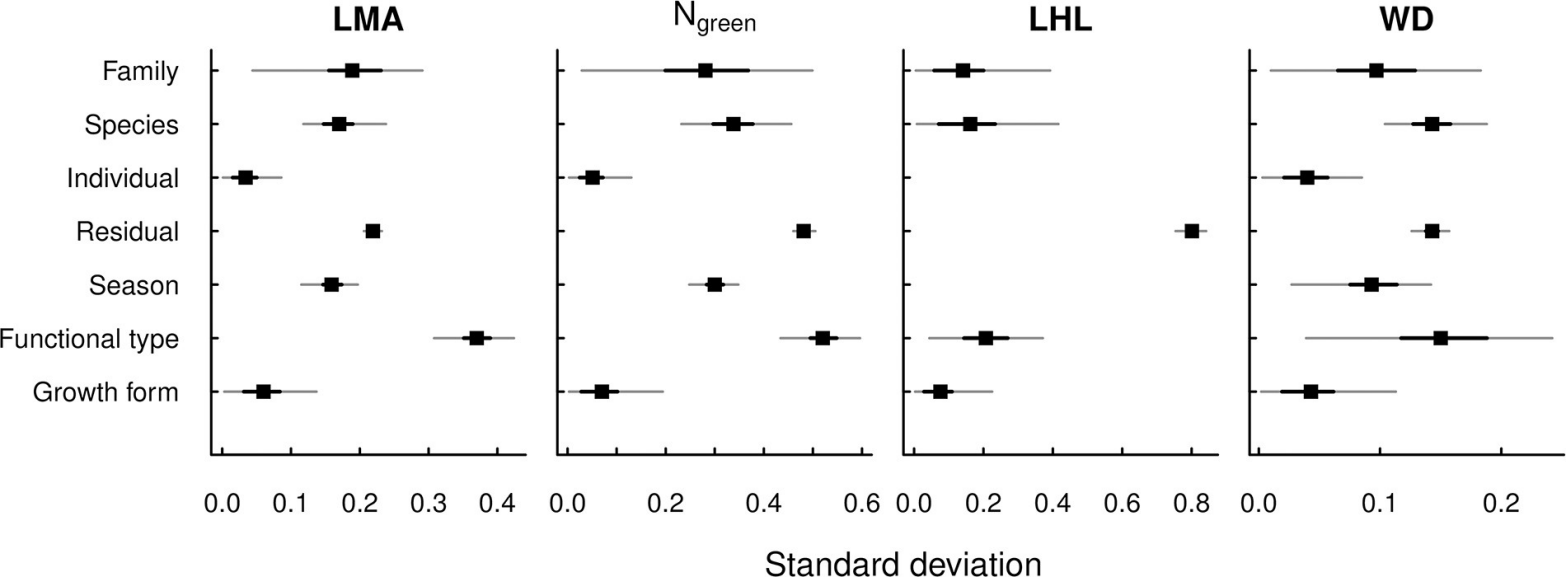
Sizes of random effects (top four values within each graph, including residual) and fixed effects (bottom three values), expressed in standard deviations (square roots of variance components), from Bayesian multilevel analyses of variance for each trait. Circles are estimated posterior means of effect sizes, short thick lines are the 50% posterior credible intervals and long thin lines are the 95% posterior credible intervals. N_green_: nitrogen concentration in green leaves; LHL: leaf half-life; LMA: leaf mass per area; WD: wood density. See text for full explanation of analysis method.

LMA, N_green_ and WD showed similar patterns of effects in the multilevel ANOVA, with functional type having the largest explanatory power, family, species and season having intermediate power (although for WD, functional type and species had similar explanatory power), growth form and variation among individuals having the lowest explanatory power (Fig 3). For LHL, functional type had similar explanatory power as family, species and growth form, yet residual variation was particularly large compared with the explanatory terms, which indicated that there was large variation within species for this trait. For example in *Camellia oleifera*, LHL ranged from 256 to 2211 days among eight individuals. The classical ANOVA results (S3(a) Table) were qualitatively very similar to multilevel ANOVA results, so only the latter are presented.

A wide range of soil nitrogen content (0.8–9.65 g/kg) and organic matter content (6–306 g/kg) indicated a large heterogeneity of abiotic conditions across our study site. However, soil nitrogen content and organic matter content explained only a very small proportion of trait variation and no significant relationship between any of the traits and soil properties could be found (test for soil nitrogen content shown in S3(b) Table).

### Trait correlations and evolutionary divergence

LMA, LHL and WD were significantly positively correlated with each other and significantly negatively correlated with N_green_ for both overall correlations and correlations between species means (Table 2). Within-species correlations were only statistically significant in N_green_–LMA. Removing the influence of common phylogenetic history by using PICs yielded almost the same results as species mean correlations (Table 2), indicating that these were not constrained by phylogeny but also that our species selection was well spread out over the entire phylogenetic tree.

**Table 2:** Correlations between traits: (1) overall correlations; (2) correlations between species means; (3) inter-specific evolutionary divergence correlations using phylogenetically independent contrasts (PICs); (4) within-species correlations. Numbers show Pearson correlation coefficient. Traits were log-transformed prior to analysis.

Trait pair	Trait correlations			
	Overall	Species mean	PIC	Within-species
N_green_–LMA	–0.781 ***	–0.779 ***	–0.745 ***	–0.615 ***
N_green_–LHL	–0.308 ***	–0.535 ***	–0.435 **	0.135
N_green_–WD	–0.371 ***	–0.564 ***	–0.605 ***	0.000
LMA–LHL	0.348 ***	0.654 ***	0.524 **	0.028
LMA–WD	0.357 ***	0.416 **	0.460 **	0.030
LHL–WD	0.279 *	0.378 *	0.380 *	–0.139

***: P < 0.001;

**: P < 0.01;

*: P < 0.05.

We found a significant phylogenetic signal in the variation of two traits, N_green_ (K = 0.767, K_rand_ = 0.368, 95% CI = 0.245–0.515) and LMA (K = 0.596, K_rand_ = 0.257, 95% CI = 0.371–0.514), indicating that these two traits were relatively well conserved within lineages (e.g. families). The two other traits, WD and LHL, showed no significant phylogenetic signal (WD: K = 0.4, K_rand_ = 0.367, 95% CI = 0.259–0.527, LHL: K = 0.445, K_rand_ = 0.436, 95% CI = 0.293–0.66). However, WD showed significant variation between genera and species within families (see S3(a) Table), indicating more recent evolutionary divergences.

### Drivers of trait covariance

**Fig 4:**
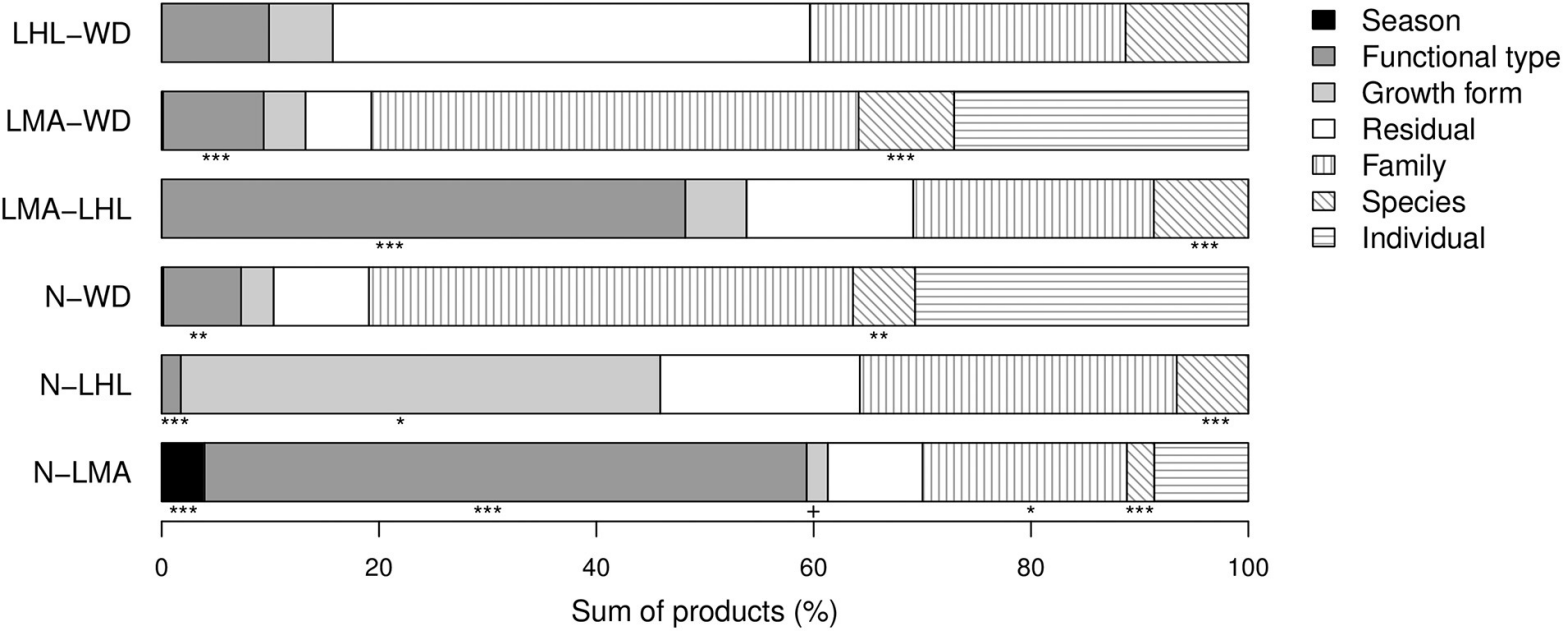
Partitioning of sums of products between pairs of traits. Sections with solid filling represent fixed effects (season, functional type and growth form), while sections with hatching lines represent random effects (family, species and individual). Sections with no shadings represent sum of products explained by residual covariance. ***: P < 0.001; **: P < 0.01; *: P < 0.05;–: P<0.05 (contributions to sums of products from negative covariance components, all other significant ones were positive). N_green_: nitrogen concentration in green leaves; LHL: leaf half-life; LMA: leaf mass per area; WD: wood density.

When the covariance among pairs of traits was partitioned, functional type explained a significant amount of the total covariance for each pair except LHL–WD (Fig 4). Growth form was less important except for the pair N_green_–LHL where it explained 44% of the total covariance. For N_green_–LMA and LMA–LHL, growth form had covariance components with different sign from the overall covariance. The covariance component for the taxonomic term family was generally large but, in agreement with the PIC analysis described in the previous section, significant only for the pair N_green_–LMA, corroborating the observation that phylogeny had a limited influence on trait correlations. However, at the lowest phylogenetic level between species, we found significant covariance components that had the same signs as the overall correlations. At individual level all covariance components were non-significant, indicating that overall trait correlations were not simply due to environmental or developmental heterogeneity among individuals within species. However, within individuals the term season had a significant covariance component for N_green_–LMA because low values for N_green_ were associated with high values for LMA in September 2006 and vice versa in April 2007.

## Discussion

We analyzed the variation of and covariation between four plant traits considered characteristic of different plant functional strategies such as the ones defining the two poles of the leaf-economics spectrum or similar trait syndromes. The variation in leaf half-life (LHL), leaf mass per area (LMA), nitrogen concentration in green leaves (N_green_) and wood density (WD) we observed among individual trees and shrubs of angiosperms of a single forest covered more than half of the global range reported [2]. Leaf seasonality explained most variation in these traits, followed by season (spring vs. summer), the taxonomic classifications family and species, and smaller contributions of growth form (tree vs. shrub) and individual. Substantial fractions of covariance between the analyzed traits were explained by leaf seasonality, family and species. Effects of family on trait covariation were rarely statistically significant, a finding that was supported by analyses using phylogenetically independent contrasts.

### Variation in functional traits

The large variation in the measured leaf and wood traits of the studied species suggested that several functional strategies co-occured in the species-rich subtropical forest studied here. Leaf seasonality explained the largest amount of variation in N_green_, LMA and WD. Deciduous species were characterized by trait values generally associated with productivity, namely high leaf nitrogen (N_green_), thin leaves (low LMA) and low wood density (WD), indicating high photosynthetic capacities and high growth rates. In contrast, evergreen species were characterized by trait values associated with persistence and slow growth, namely thick, nitrogen-poor leaves (low N_green_ and high LMA) and dense wood (high WD). The strategies reflected by these trait syndromes appear fairy universal and are now conceptualized in the leaf-economics spectrum [2, 36].

**Fig 5:**
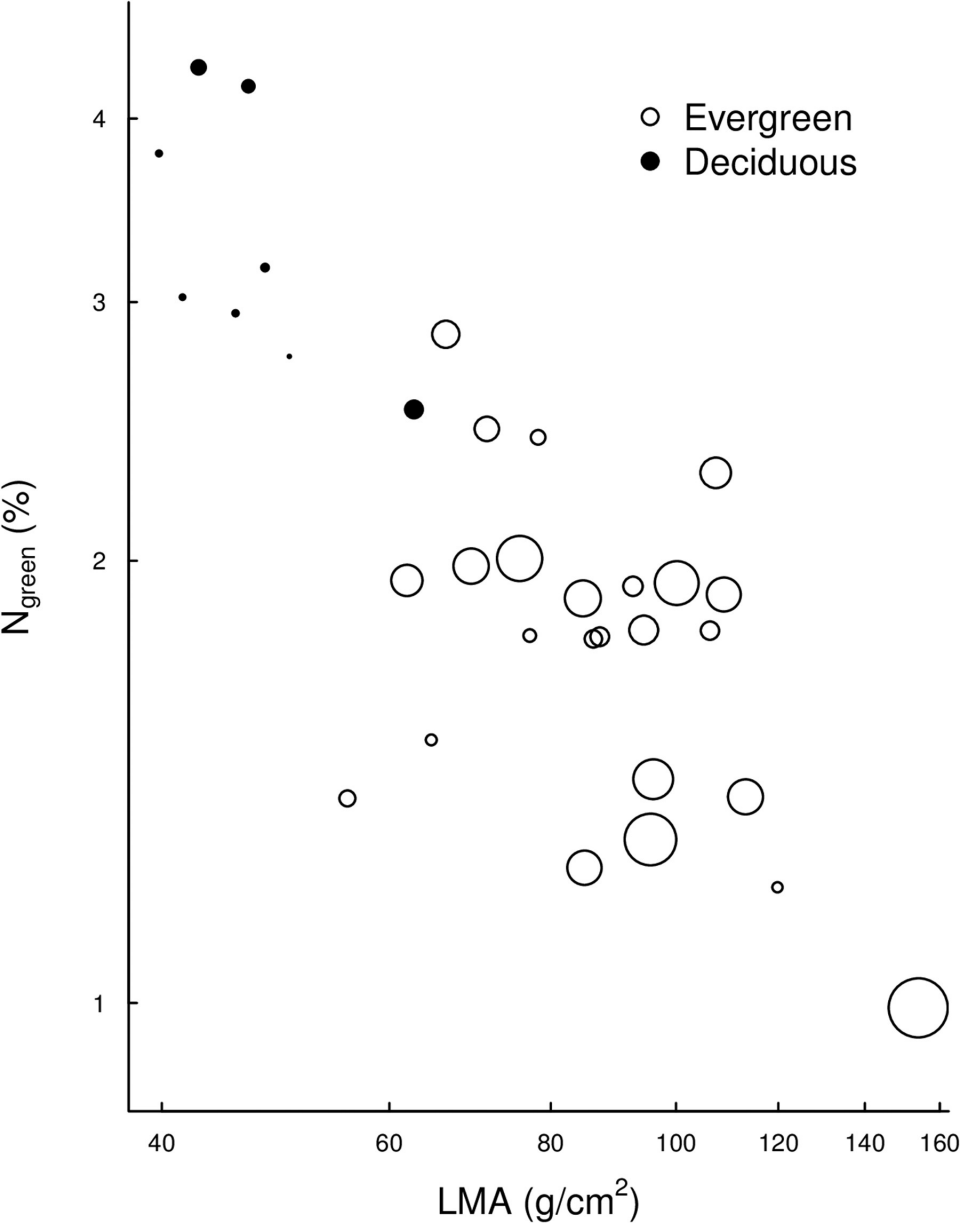
Correlation between species means of N_green_ and leaf mass per area (LMA). Circles represent evergreen species and solid points represent deciduous species. The sizes of circles and points indicate the mean leaf half-lives of the species.

In our study, leaf longevity (quantified as leaf half-life) was less clearly associated with the trait syndrome indicative of persistence than was leaf seasonality. The underlying reason was that some evergreen species maintained evergreen leaf crowns despite possessing short-lived leaves even when compared to evergreen species. For example, the evergreen species *Ilex chinensis* had shorter-lived leaves (LHL of 197 days) than the deciduous species *Rhus chinensis* (LHL of 259 days). It has been hypothesized that evergreen species with such a high turnover of short-lived leaves would share the trait syndrome for persistence of deciduous species [1]. However, our results indicate that this was not the case: The two functional types deciduous and evergreen were clearly separated along the bivariate LMA–N_green_ trait space (Fig 5), despite overlapping leaf life span (Fig 2). The only exception where trait syndrome coincided with LHL rather than with leaf seasonality was the deciduous species *Quercus variabilis* with a relatively long LHL of 331 days and a trait syndrome associated with low productivity typical of evergreen species. Leaf life spans are rarely estimated by demographic analysis for a large number of species as we did here because such measurements are very labour intensive (but see [36]. However, using deciduousness or maximum leaf age as indicators of “leaf life span” may yield traits that reflect leaf seasonality rather than true leaf life span and therefore also be more closely associated with the proposed trait syndromes.

The sorting of trait syndromes along environmental gradients can be understood in the light of “environmental filtering” narrowing the range of traits associated with successful performance in particular habitats or stages of community development. Communities in early secondary successional stages, for example, are generally dominated by productive strategies reflecting abundant resource supply and high growth rates; in contrast, communities of late successional stages are dominated by persistence strategies indicating resource scarcity and high levels of competition [7, 37]. In our study, and quite typically so for forest communities, species with different trait syndromes co-occurred. This may be explained by limiting similarity [38]. Forests are heterogeneous environments with, for example, large gradients in light availability, in particular after disturbance. Seedlings and saplings emerging in gaps clearly are at advantage adopting a fast growth–low persistence strategy, whereas adult and smaller understory species will be subject to intense competition and resource scarcity and thus only be successful with a slow growth–high persistence strategy. Simulation studies have shown that multiple species coexist in such a situation because of trade-offs between growth strategies, and only so if their niches are sufficiently different [39]—i.e. only if they differ in terms of time and space under which they become limited by particular resources. Similar coexistence of distinct trait-syndromes have also been described in other studies, including an old-field in which species with productive and persistent leaves coexisted (*Aster lanceolatus* and *Solidago canadensis*, respectively; [40]), a pattern that could be explained by complementary forms of clonal spread [41].

Functional trait values were strongly season-dependent: often the variance component quantifying the strength of seasonality effects was as large (LMA, WD) or larger (N_green_) than the variance component measuring interspecific variation. This demonstrates that the measured traits are not very rigid and that even a single species or tree individual can shift its strategy to a certain extent, e.g. from assimilate acquisition in spring to storage in autumn [42, 43]. For example, greater N_green_ in April 2007 could reflect large carbon assimilation rates at the beginning of the growing season, whereas lower leaf N contents in September 2006 might indicate a reduced demand due to a cessation of growth towards the end of the growing season. The pronounced seasonal dynamics of traits underlines the importance of considering the time of trait assessment and that the same leaf trait reflect different aspects of the functional ecology of a species depending on when it is measured. The variation of traits among conspecifics was small compared to seasonal changes, indicating that seasonally standardized species means captures the relevant functional variation in leaf traits in a community sufficiently well, even when few individuals are sampled (but see [12]).

Our analysis of taxonomic and phylogenetic contributions to variation in leaf traits revealed a high degree of phylogenetic niche conservatism for LMA and N_green_—these traits also showed the clearest effects of family in the univariate analyses of variance (Fig 1 and S3 Table). This implies that these two traits remained relatively distinct between groups of species deriving from different common ancestors (families), even though differences among species within each group also were statistically significant. The two other functional traits, LHL and WD, showed no phylogenetic signal, yet at least for the latter differences between genera and species within families were statistically significant (S3(a) Table). The effects of species (and for WD also genera) indicate recent evolutionary divergence events [44, 45]. In contrast, our analysis showed that LHL varied most “freely” possibly indicating that leaf life span depends less on (phylo)genetical determinants. This may be different for deciduousness or maximum leaf longevity. However, clearly tree growth will depend more on actual than on maximum leaf life span and we therefore maintain that LHL is a preferable metric in studies aiming at relating leaf functional traits to plant performance.

### Patterns of covariation in functional traits

The strong correlations among N_green_, LMA and LHL found in the studied forest community (Table 2) is consistent with results from global compilations of data across different ecosystem types and plant functional groups, including herbaceous and woody species [2, 46]. However, in our study, the slopes of bivariate trait relationships between N_green_−LMA (–1.06, 95% CI: –0.88– –1.29) and LHL–LMA (2.54, 95% CI: 1.92–3.36) were significantly higher than those in the global dataset of [2] (–0.78, 95% CI: –0.76– –0.81 for N_green_−LMA and 1.71, 95% CI: 1.61–1.81 for LL−LMA; see Fig 5). This indicates that in our forest community N_green_ decreased and LHL increased more rapidly with increasing LMA than expected based on the global patterns across sites. The reason for this difference may be that our study only included trees and shrubs with generally low LMA [2].

As for leaves, trait syndromes have also been identified for stem [11] and root traits [47]. An important question is whether these trait syndromes vary independently or whether these are coordinated, reflecting fundamental ecological strategies. The relatively strong correlations between the only stem trait, wood density (WD), and the three leaf traits N_green_, LMA and LHL suggest that the observed trait syndromes can be extended to a leaf-stem economics. Such a trait coordination across organs is supported by many [48–53] studies, although there are exceptions [12, 46, 54, 55]. Reich [56] has argued that such a coordinated change in traits reflects a whole-plant strategy of “fast” vs. “slow” growth. Specifically, a greater WD is considered to be related to a lower growth rate, higher survival, higher mechanical strength and higher resistance to herbivory [11]. Leaves with high LMA and wood with high WD are expensive to build but enable plants to withstand physical and herbivore damage. The high LMA of expensive leaves reduces photosynthetic capacity by diluting the proportion of leaf tissue allocated to photosynthetic enzymes (reflected in lower leaf nitrogen concentration) and can lead to CO_2_ diffusion limitation [1, 57]. The smaller vessel diameters in dense wood can reduce xylem conductivity and thus affect plant traits regulating photosynthesis [51]. Species adopting a contrasting strategy can be expected to have leaves with low LMA that are cheap to build and have a large light-capturing surface per unit of assimilates invested. To maximize growth rates, such species would tend to have lower WD and at the same time higher xylem conductivity and photosynthetic capacity. As a trade-off, they would be more susceptible to physical and herbivore damage.

Our analyses of the patterns of covariation in functional traits across the studied woody species in a subtropical forest in China showed that trait coordination was associated with plant functional type and additionally with plant family and species. That is, phylogenetic and genetic constraints to a certain extent forced the different traits to covary, which was also reflected in the significant inter-specific evolutionary divergence correlations using phylogenetically independent contrasts. This trait coordination underlies the emergence of the trait syndromes discussed above in relation to different functional strategies and it prohibits others that would be conceivable in the absence of the observed patterns of covariation, for example the combination of high N_green_ and high LMA or the combination of low N_green_ and low LMA. While the latter should be rapidly eliminated by selection due to both slow growth and low persistence [58], the former would constitute a “Darwinian demon” that obviously has not evolved due to inherent trade-offs. The smaller and non-significant covariance components associated with individual (see Fig 3) indicate that at the within-species level the two combinations low N_green_ and low LMA or high N_green_ and high LMA as well as other trait combinations are not impossible and may reflect particular local conditions. However, we were unsuccessful in relating trait variation among individuals to measured soil heterogeneity.

## Conclusion

Functional trait variation and covariation among species across spatial scales has been studied intensively in the past to gain insight into plant adaptations to the environment. However, a large proportion of the inter-specific variance in traits, e.g. 36% in SLA and 38% in leaf nitrogen per mass, is found at the local scale within communities [2]. We found that despite substantial heterogeneity in time and space, plant functional type and species were the major sources of trait variation. When considering trait-pair correlations, we found again plant functional type and species to be the most important sources of covariance. Species could be placed along a spectrum—similar to the leaf or stem-leaf economics spectrum reported from different communities—which runs from deciduous species with thin leaves, high leaf nitrogen concentration and light wood to evergreen species with thick leaves, low leaf nitrogen concentration and dense wood. Actual leaf life spans vary more freely than suggested by the strict difference between deciduous vs. evergreen functional types and between species within functional types and may thus strongly affect differences in tree growth at the within-species level. The trait syndromes we observed are maintained by strong inter-specific correlations whereas smaller within-species covariance components allow individual trees to deviate from the interspecific trait coordination and thus respond flexibly to environmental heterogeneity or other factors.

## Supporting information

S1 Table Posterior means, standard deviations and credible intervals of the effect sizes from Bayesian multilevel analyses of variance for each of the four measured traits. Rhat shows how well convergence was achieved (the closer to 1 the better). N_green_: nitrogen concentration in green leaves; LMA: leaf mass per area; LHL: leaf half-life; WD: wood density; GF: growth form; FT: functional type.
(PDF)

S2 Table Species mean (standard deviation) of investigated traits. N_green_: nitrogen concentration in green leaves; LMA: leaf mass per area; LHL: leaf half-life; WD: wood density.
(PDF)

S3 Table ANOVA of traits without (a) or with soil nitrogen as covariate (b). N_green_: nitrogen concentration in green leaves; LMA: leaf mass per area; LHL: leaf half-life; WD: wood density; Df.: degree of freedom; %SS: percent contribution to total sum of squares; P: level of significance; LogNsoil: soil nitrogen content (log scale).
(PDF)
