# Supplementary material for: Decomposing functional trait associations in a Chinese subtropical forest
Source: PLoS One. 2017 Apr 18;12(4):e0175727. doi: 10.1371/journal.pone.0175727 (PMC5395190; doi:10.1371/journal.pone.0175727)
Supplement: S1 Table — Rhat shows how well convergence was achieved (the closer to 1 the better). Ngreen: nitrogen concentration in green leaves; LMA: leaf mass per area; LHL: leaf half-life; WD: wood density; GF: growth form; FT: functional type. (PDF) [file pone.0175727.s001.pdf]

S1 Table. Posterior means, standard deviations and credible intervals of the effect sizes from Bayesian multilevel analyses of variance for each of the four measured traits. Rhat shows how well convergence was achieved (the closer to 1 the better). N_{green}: nitrogen concentration in green leaves; LMA: leaf mass per area; LHL: leaf half-life; WD: wood density; GF: growth form; FT: functional type.

Trait	Source	Mean	SD	2.50%	25%	50%	75%	97.50%	Rhat
LMA	GF	0.060	0.036	0.003	0.031	0.057	0.084	0.137	1.004
	FT	0.370	0.029	0.308	0.351	0.371	0.390	0.424	1.000
	Season	0.159	0.021	0.115	0.146	0.161	0.174	0.197	1.005
	Residual	0.219	0.007	0.206	0.215	0.219	0.223	0.232	1.001
	Individual	0.034	0.024	0.001	0.015	0.031	0.050	0.086	1.001
	Species	0.170	0.031	0.118	0.147	0.167	0.190	0.238	1.018
	Family	0.189	0.060	0.044	0.155	0.196	0.231	0.291	1.013
N_{green}	GF	0.070	0.053	0.003	0.028	0.060	0.102	0.194	1.007
	FT	0.520	0.041	0.434	0.495	0.522	0.548	0.596	1.001
	Season	0.300	0.025	0.248	0.283	0.301	0.317	0.348	1.005
	Residual	0.481	0.011	0.460	0.474	0.481	0.489	0.505	1.003
	Individual	0.051	0.034	0.003	0.025	0.045	0.072	0.130	1.007
	Species	0.338	0.058	0.232	0.297	0.335	0.378	0.456	1.004
	Family	0.281	0.122	0.029	0.199	0.291	0.368	0.499	1.011
WD	GF	0.043	0.030	0.002	0.019	0.039	0.062	0.113	1.001
	FT	0.150	0.052	0.039	0.117	0.156	0.188	0.242	1.000
	Season	0.093	0.029	0.027	0.075	0.096	0.114	0.142	1.003
	Residual	0.143	0.008	0.126	0.138	0.143	0.148	0.157	1.001
	Individual	0.040	0.023	0.003	0.021	0.038	0.057	0.085	1.001
	Species	0.143	0.022	0.104	0.127	0.142	0.158	0.188	1.000
	Family	0.097	0.045	0.010	0.065	0.098	0.129	0.183	1.001
LHL	GF	0.076	0.061	0.003	0.029	0.061	0.109	0.225	1.002
	FT	0.207	0.086	0.044	0.144	0.209	0.270	0.371	1.004
	Residual	0.801	0.022	0.754	0.788	0.802	0.814	0.842	1.001
	Species	0.162	0.112	0.008	0.071	0.145	0.234	0.416	1.004
	Family	0.141	0.105	0.005	0.058	0.121	0.200	0.392	1.001
