# Supplementary material for: Decomposing functional trait associations in a Chinese subtropical forest
Source: PLoS One. 2017 Apr 18;12(4):e0175727. doi: 10.1371/journal.pone.0175727 (PMC5395190; doi:10.1371/journal.pone.0175727)
Supplement: S2 Table — Ngreen: nitrogen concentration in green leaves; LMA: leaf mass per area; LHL: leaf half-life; WD: wood density. (PDF) [file pone.0175727.s002.pdf]

S2 Table. Species mean (standard deviation) of investigated traits. N_{green}: nitrogen concentration in green leaves; LMA: leaf mass per area; LHL: leaf half-life; WD: wood density.

Family	Genus	Species	LMA (g/cm^2)	N_{green} (%)	LHL (days)	WD (g/cm^3)
Anacardiaceae	*Choerospondias*	*axillaris*	45(23)	2.5(0.7)		44(3.2)
	Pistacia	*chinensis*	68(18)	2.4(0.4)		71.5(13.9)
	Rhus	*chinensis*	43(3)	4.3(0.5)	260	44.2(3.7)
		punjabensis	42	3	98	51.9
	Toxicodendron	*succedaneum*	45(14)	3.2(0.8)		49.8(5.1)
Aquifoliaceae	*Ilex*	*chinensis*	120(28)	1.2(0.2)	185(35)	62.9(10.7)
		szechwanensis	85(10)	1.2(0.1)	619(312)	
Araliaceae	*Aralia*	*chinensis*	40(6)	3.8(0.2)	107	44.3(5.9)
	Kalopanax	*pictus*	59(24)	2.9(0.7)		46.4(9.8)
Betulaceae	*Betula*	*luminifera*	48(14)	3.2(0.9)	137	48.5(4.9)
Cornaceae	*Cornus*	*controversa*	46(16)	2.9(0.4)	111	42.3(1.1)
Ebenaceae	*Diospyros*	*kaki*	40(18)	2.6(1.2)		67.2
Elaeocarpaceae	*Elaeocarpus*	*japonicus*	93(13)	1.9(0.3)	351(87)	53.9(12.9)
Euphorbiaceae	*Mallotus*	*philippensis*	71(14)	2.5(0.2)	440(327)	62.8(3.1)
		tenuifolius	47(17)	4.2(1.2)	223	42(12.9)
Fagaceae	*Castanopsis*	*carlesii*	80(11)	1.8(0.1)		68.4(13.4)
		fargesii	87(11)	1.8(0.1)	335(243)	60.5(17.6)
		sp.	89(9)	1.8(0.2)		62.8(9.9)
	Cyclobalanopsis	*glauca*	85(12)	1.9(0.3)	650	68.4(10.7)
	Lithocarpus	*hancei*	86(12)	1.8(0.2)	314(142)	60(9.1)
	Quercus	*serrata*	50(18)	2.8(0.8)	56	69.8(6.2)
		variabilis	63(20)	2.5(0.6)	317(124)	76.7(12.6)
Juglandaceae	*Platycarya*	*strobilacea*	52(13)	2.9(0.9)		52.1(3.9)
	Pterocarya	*stenoptera*	42(7)	3.1(0.2)		36.2(2.7)
Lauraceae	*Cinnamomum*	*bodinieri*	100(14)	1.9(0.2)	791(39)	52.8(3.6)
	Lindera	*communis*	94(14)	1.8(0.4)	522(236)	
	Machilus	*pingii*	106(20)	1.8(0.2)	330	49.4(1.9)
Moraceae	*Ficus*	*henryi*	78(16)	2.4(0.4)	262(116)	48.3(7.5)
		heterophylla	47(18)	2.4(1.1)		68.4
Myrsinaceae	*Myrsine*	*africana*	64(13)	1.5(0.1)	195(40)	
Olacaceae	*Schoepfia*	*jasminodora*	66	2.9(0.7)	488	71.2(22.8)
Pittosporaceae	*Pittosporum*	*podocarpum*	62(11)	1.9(0.2)	567(382)	72.1
		sp.	69(12)	2(0.2)	639(114)	76.7(22.8)
Rosaceae	*Photinia*	*davidsoniae*	56	1.4(0.2)	288(258)	74.6
	Pyracantha	*fortuneana*	75(12)	1.4(0.2)		
Rutaceae	*Zanthoxylum*	*ovalifolium*	107(20)	2.3(0.3)	551(238)	72.3(4.9)
Symplocaceae	*Symplocos*	*anomala*	77(19)	1.8(0.3)	225(100)	60.5(6.3)
		cochinchinensis var. *laurina*	113(14)	1.4(0.2)	634(532)	72.7(26.6)
		paniculata	42(6)	2.6(0.1)		58.8(5.7)
		stellaris	109(21)	1.9(1)	613(570)	55.8(6.9)
		sumuntia	76(14)	2(0.3)	819(604)	55.7(3.5)
Theaceae	*Camellia*	*oleifera*	154(25)	1(0.2)	1069(682)	69.2(6.6)

	Eurya	*alata*	96(13)	1.3(0.2)	934(433)	61.6(8.9)
		nitida	96(13)	1.4(0.2)	715(300)	60.1(2.6)
Ulmaceae	*Celtis*	*vardervoetiana*	30(8)	3.5(1.4)		51.4(4.7)
