# Supplementary material for: Decomposing functional trait associations in a Chinese subtropical forest
Source: PLoS One. 2017 Apr 18;12(4):e0175727. doi: 10.1371/journal.pone.0175727 (PMC5395190; doi:10.1371/journal.pone.0175727)
Supplement: S3 Table — Ngreen: nitrogen concentration in green leaves; LMA: leaf mass per area; LHL: leaf half-life; WD: wood density; Df.: degree of freedom; %SS: percent contribution to total sum of squares; P: level of significance; LogNsoil: soil nitrogen content (log scale). (PDF) [file pone.0175727.s003.pdf]

S3 Table. ANOVA of traits without (a) or with soil nitrogen as covariate (b). N_{green}: nitrogen concentration in green leaves; LMA: leaf mass per area; LHL: leaf half-life; WD: wood density; Df.: degree of freedom; %SS: percent contribution to total sum of squares; P: level of significance; LogNsoil: soil nitrogen content (log scale).

(a)

	LMA			N_{green}		
	Df.	%SS	P	Df.	%SS	P
Season	1	0.24	0.086	1	2.43	0.000
FT	1	38.34	0.000	1	45.52	0.000
GF	1	0.15	0.526	1	2.03	0.007
Family	19	17.22	0.029	19	20.29	0.021
Genus	13	4.14	0.590	13	4.50	0.157
Species	11	3.97	0.000	11	2.06	0.002
Individual	218	17.96	0.434	211	14.02	0.999
Residual	223	17.97		75	9.16	
Total	487	100.00		332	100.00	

	LHL			WD		
	Df.	%SS	P	Df.	%SS	P
Season				1	0.16	0.325
FT	1	14.14	0.015	1	7.49	0.002
GF	1	8.58	0.041	1	1.22	0.115
Family	15	17.85	0.262	18	27.09	0.805
Genus	7	5.10	0.788	11	25.77	0.005
Species	7	9.57	0.110	10	4.10	0.015
Individual				148	26.16	0.349
Residual	57	44.76		50	8.00	
Total	89	100.00		240	100.00	

(b)

	LMA			N_{green}		
	Df.	%SS	P	Df.	%SS	P
LogNsoil	1	0.37	0.192	1	0.54	0.108
Season	1	0.23	0.255	1	1.97	0.021
FT	1	32.81	0.000	1	40.98	0.000
GF	1	0.01	1.000	1	1.30	0.472
Family	18	27.82	0.088	17	28.75	0.075
Genus	6	3.61	0.457	7	1.29	0.922
Species	7	3.92	0.020	7	4.01	0.013
Individual	55	11.71	0.210	51	10.20	0.950
Residual	110	19.52		33	10.98	
Total		100.00			100.00	

	LHL			WD		
	Df.	%SS	P	Df.	%SS	P
LogNsoil	1	1.26	0.366	1	1.24	0.131
Season				1	0.28	0.458
FT	1	12.60	0.084	1	10.08	0.035
GF	1	15.62	0.006	1	5.20	0.321
Family	12	20.69	0.274	16	30.90	0.288
Genus	3	3.54	0.739	6	18.13	0.159
Species	5	13.65	0.146	4	4.13	0.117
Individual				38	19.79	0.447
Residual	22	32.63		21	1.24	
Total		100.00			100.00	
